# Genetic subclonal complexity and miR125a-5p down-regulatio identify a subset of patients with inferior outcome in low-ris CLL patients

**DOI:** 10.18632/oncotarget.1382

**Published:** 2013-10-30

**Authors:** Gian Matteo Rigolin, Elena Saccenti, Lara Rizzotto, Manuela Ferracin, Sara Martinelli, Luca Formigaro, Francesca Cibien, Maurizio Cavallari, Enrico Lista, Giulia Daghia, Olga Sofritti, Maria Ciccone, Francesco Cavazzini, Laura Lupini, Cristian Bassi, Barbara Zagatti, Massimo Negrini, Antonio Cuneo

**Affiliations:** ^1^ Hematology Section, Department of Medical Sciences, University of Ferrara, University Hospital Arcispedale S. Anna, Ferrara, Italy; ^2^ Laboratory for Technologies of Advanced Therapies (LTTA) and Department of Morphology, Surgery and Experimental Medicine, University of Ferrara, Ferrara, Italy

**Keywords:** Oncotargets, miR-125a-5p, CLL, CD38, prognosis

## Abstract

The majority of patients with chronic lymphocytic leukemia (CLL) and favorable prognostic features live for long periods without treatment. However, unexpected disease progression is observed in some cases. In a cohort of untreated CD38-CLL patients with normal FISH or isolated 13q− we found that, by fluorescence in situ hybridization (FISH), 16/28 cases presented, within immunomagnetic sorted CD38+ cells, genetic lesions undetectable in the CD38- fraction. These patients showed a shorter time to first treatment (TTFT, p=0.0162) in comparison to cases without FISH lesions in CD38+ cells. Patients with FISH abnormalities in CD38+ cells showed a distinctive microRNA profile, characterized by the down-regulation of miR-125a-5p both in the CD38- and CD38+ populations. In an independent cohort of 71 consecutive untreated CD38- CLL with normal FISH or isolated 13q−, a lower miR125a-5p expression was associated with a shorter TTFT both in univariate and multivariate analysis (p=0.003 and 0.016, respectively) and with a higher prevalence of mutations (7/12 vs 0/8, p=0.015) as assessed by next-generation sequencing. In conclusion, our data showed previously unrecognized subclonal heterogeneity within the CD38+ fraction of CD38- CLL patients with low-risk FISH findings and suggested an association between down-regulated miR-125a-5p expression, genetic complexity and worse outcome.

## INTRODUCTION

Chronic lymphocytic leukemia (CLL) is a clinically heterogeneous disease [1-3]. Several adverse prognostic features have been identified including stage [4], CD38 positivity [5], the unmutated configuration of the variable region of the immunoglobulin heavy chain gene (*IGHV*) [5], ZAP70 positivity [6], chromosome aberrations [7, 8] and molecular abnormalities [9].

Even though most of CLL patients with favorable prognostic features, i.e. CD38-, mutated IGHV, absence of chromosome lesions or isolated 13q−, live for long periods without any treatment, some cases may show progression to a more aggressive leukemia. The biologic and molecular characteristics predicting disease progression in these patients are unknown.

CD38 is considered as a dynamic indicator of cell activation and proliferation that may prelude clonal evolution and worse clinical outcome [10]. Interestingly, by representational oligonucleotide microarray analysis (ROMA), copy number differences between CD38+ and CD38- cells were documented in some patients [11] while the heterogeneity in the subclonal architecture of the leukemic cells was suggested to correlate with a poor clinical outcome [12]. Moreover, in CLL distinct gene expression profiles were associated to CD38 expression [13] and a unique microRNA expression signature was shown to be associated with activation markers and unfavorable prognostic factors [14].

In order to better understand the biologic and molecular features predicting disease progression in CLL patients with favorable prognostic features we designed a two-phase study having the following aims: phase 1, a) to assess whether genetic lesions may be present in the minority of CD38+ cells in a series of untreated low-risk CLL patients as defined by CD38 negativity (CD38+ cells < 7%) and favorable genetic findings, b) to identify biologic factors associated with genetic lesions in the small CD38+ fraction of CD38- CLL patients and predicting for disease progression; phase 2, c) to validate our findings in an independent cohort of consecutive untreated CD38-CLL patients with favorable FISH findings.

## RESULTS

### Patients

In this study 2 cohorts of patients have been considered. Cohort one (C1) included 28 untreated CLL patients seen between 2005 and 2006. Cohort two (C2) consisted of 71 consecutive untreated CLL patients diagnosed between 2007 and 2011. The principal clinical characteristics of C1 and C2 are reported in Table 1.

**Table 1:** Principal clinical and biologic characteristics of the patients of the cohort 1 and cohort 2

	Cohort 1	Cohort 1
N of patients	28	71
M/F	16/12	49/22
Age mean yrs (range)	65 (50-91)	64 (38-86)
Stage (Binet) a/b/c	28/0/0	63/8/0
FISH neg/13q deletion	14/14	40/31
ZAP70 (>30%) neg/pos	22/5	61/10
IGHV mut/unmut	20/2	60/11
TP53 mut/unmut	0/18	0/69

### FISH analysis on immunomagnetically sorted cells in patients of C1

Results of FISH analysis in CD38- cells and CD38+ cells in C1 are reported in table 2. In 16/28 patients, genetic aberrations were detected in CD38+ cells and not in C38- cells. 11q deletion was seen in 9 cases, 13q deletion in 8 cases, 17p deletion in 8 cases, 14q32 rearrangements in 7 cases, trisomy 12 in 4 cases, biallelic 13q deletion in 2 cases (table 2). In the remaining 12 patients no additional genetic lesions were found in the CD38+ population as compared to the CD38- cells. Hybridization patterns with the control probes were within the expected normal range.

**Table 2:** FISH results in CLL patients with detectable genetic lesions in CD38+ cells (*bilallelic 13q deletion)

	FISH results on CD38- cells(%)	FISH results on CD38+ cells (% of positive cells)						
case		del(13q)	del(11q)	Trisomy 12	14q32 rearr	del(17p)	Number of additional lesions in CD38+ cells	Cohybridization
56	13q del (20%)	25	20	Neg	32	24	3	Different cells
58	13q del (30%)	28	30	Neg	28	18	3	Different cells
41	Neg	23	24	Neg	Neg	40	3	Different cells
50	13q del (60%)	37	21	15	Neg	33	3	ND
46	13q del (18%)	42	22	Neg	Neg	38	2	ND
49	13q del (60%)	37	23	Neg	21	20	3	Different cells
61	Neg	33	26	Neg	22	23	3	Different cells
43	Neg	34	Neg	Neg	27	Neg	2	Same cells
56	Neg	19	Neg	Neg	49	Neg	2	Different cells
63	Neg	20 (69*)	Neg	Neg	Neg	21	2	ND
45	13q del (69%)	58	33	17	Neg	Neg	2	Different cells
54	Neg	62*	Neg	Neg	18	Neg	2	Same cells
60	Neg	18	Neg	Neg	Neg	Neg	1	NA
48	13q del (38%)	45	20	Neg	Neg	Neg	1	Different cells
57	13q del (30%)	37	Neg	19	Neg	Neg	1	ND
64	Neg	34	Neg	33	Neg	Neg	1	Same cells
								

**Figure 1:**
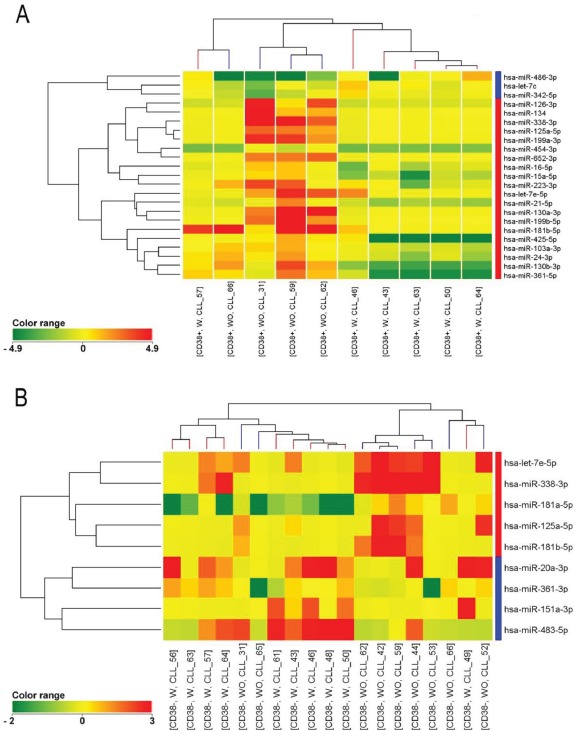
Cluster analysis of patients with (W) and without (WO) lesions in the CD38+ fraction miRNA profiling of CD38+ (a) and CD38- (b) cells from CD38- CLL patients with (red) and without (blue) FISH lesions in the CD38+ fraction. A distinctive miRNA profile characterized patients with and without FISH lesions both in the CD38+ (23 microRNAs) and CD38- cells (9 microRNAs). The colors of the genes represented on the heatmap correspond to the expression values normalized on miRNA mean expression across all samples: green indicates down-regulated; red indicates up-regulated.

To assess whether the genetic lesions were on different clones, cohybridization experiments using appropriate probes were performed in 11 cases with >1 aberration in CD38+ cells. In these experiments it was shown that the genetic lesions involved different CD38+ cells in 8 cases and involved the same cells in the remaining 3 cases (Figure 1 supplemental).

### miRNA profiling on immunomagnetically sorted cells in C1

**Figure 2:**
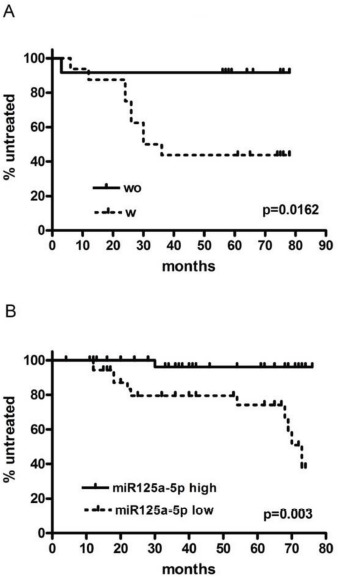
Time to first treatment in cohort 1 (A) and cohort 2 (B) respectively, according to the presence (W) or not (WO) of FISH lesions in CD38+ cells and to the level of expression of miR125a-5p (low or high), respectively

We evaluated the global miRNA expression profile of 19 patients by considering CD38+ and CD38- cell populations separately. We found that at diagnosis most of the patients with genetic lesions in CD38+ cells (W) had a distinctive miRNA profile when compared to those without genetic lesions (WO), both in the CD38+ (Figure 1A) and CD38- subpopulation (Figure 1B). Twenty-three miRNAs were found to be differentially expressed in CD38+ population (corrected p<0.05, Table 1 supplemental) and 9 miRNAs were found to be differentially expressed in CD38- population (corrected p<0.05, Table 2 supplemental). Four miRNAs were found to be down-regulated in patients with vs. without genetic aberrations in CD38+ cells both in the CD38+ and CD38- populations: let-7e-5p, miR-125a-5p, miR-181b-5p and miR-338-3p. Interestingly, miR-125a-5p showed the higher degree of significance both in CD38+ and CD38- subpopulations and was therefore chosen for further clinical correlations. The down-regulation of miR-125a-5p was confirmed by RT-qPCR analysis (Figure 2 supplemental).

### Clinical outcome

In C1, the presence of additional FISH abnormalities in the CD38+ cells correlated with a more aggressive course of the disease that was characterized by a shorter TTFT (HR 8.052, range 1.332-16.760, p=0.0162, Figure 2a). No difference was instead observed between the 2 groups of patients concerning the principal clinical and biological characteristics (Table 3).

**Table 3:** Clinical and biological characteristic of the patients in cohort 1 and cohort 2 according to the presence/absence of additional FISH lesions in CD38+ cells and the level of miR-125a-5p expression, respectively

	Cohort 1				Cohort 2		
	Patients with FISH lesions	Patients without FISH lesions	P		miR-125a low	miR-125a high	p
N of patients	16	12	-		35	36	-
M/F	9/7	7/5	ns		26/9	23/13	ns
Age mean yrs (range)	64 (50-91)	66 (52-80)	ns		64 (50-91)	66 (52-80)	ns
Binet stage a/b/c	16/0/0	12/0/0	ns		30/4/1	33/3/0	ns
ZAP70 (>30%) pos/neg	4/12	1/10	ns		6/29	4/32	ns
FISH neg/13qdeletion	8/8	6/6	ns		16/19	16/20	ns
IGHV mut/unmut	11/2	9/0	ns		30/5	30/6	ns
Treated / untreated	9/7	1/11	0.0159		11/24	1/35	ns

Having shown that miR-125a-5p down-regulation was strongly associated with additional FISH lesions on CD38+ cells and with shorter TTFT, we investigated in an independent cohort of 71 consecutive untreated low-risk CLL the clinical relevance of miR125a-5p expression. For the purposes of this analysis patients were subdivided into 2 groups based on the 50^th^ percentile of miR125a-5p distribution (range 0.00276 – 6.57599 ΔΔCq, 50^th^ percentile 0.85082 ΔΔCq). The median follow up was 43 months and no difference was observed between the 2 groups of patients in terms of clinical and hematologic characteristics (Table 3). However, patients with a lower miR125a-5p expression were characterized, by a more aggressive course of the disease and a shorter TTFT both in univariate and in multivariate analysis (Table 4 and figure 2b).

### Mutational analysis by next generation sequencing (NGS)

Because down-regulation of miR125a-5p was associated with the presence of genetic lesions in a minor CD38+ fraction of the total neoplastic cells population in C1, we screened 20 consecutive patients in C2 by mutational analysis using next-generation sequencing detecting somatic mutations in minor cell fractions (sensitivity 5%) [15]. Seven out of 12 patients with low miR-125a-5p expression displayed mutations in the CLL population (table 5) as compared with no patients out of 8 cases with high miR-125a-5p expression (7/12 vs 0/8, p=0.015).

## DISCUSSION

In CLL patients, there is evidence that a complex subclonal architecture of the leukemic clone may correlate with a more aggressive course of the disease and that in most cases genomic abnormalities are recurrent non-random events that expand over time due to a Darwinian selective pressure [11, 16].

CD38 is a marker of unfavorable prognosis and an indicator of cell activation and proliferation that may prelude clonal evolution and ultimately a worse clinical outcome [10]. Indeed CLL is a disease in which the host's microenvironment promotes leukemic cell growth, leading to sequential acquisition and accumulation of genetic alterations [11]. Interestingly, there is also evidence that proliferation centers are enriched in CD38+ cells and that a high frequency of genetic lesions may accumulate at these sites [17].

**Table 4:** Factors affecting TTFT in univariate and multivariate analysis in the cohort 2

variable	N of patients	Univariate analysis		Multivariate analysis	
		HR (95% CI)	p	HR (95% CI)	P
IGHV unmut/mut	11 vs 60	3.939 (1.66-66.97)	0.012	5.51 (1.40-21.63)	0.015
MiR-125a-5p high/low	36 vs 35	11.45 (1.80-17.60)	0.003	13.17 (1.40-21.63)	0.016
Stage b-c/a	8 vs63	3.84 (1.29-87.40)	0.028	3.52 (0.88-14.131)	0.076
ZAP70 pos/neg	10 vs 61	1.45 (0.27-8.98)	ns	-	-
FISH normal /13q deletion	32 vs 39	0.97 (0.30-3.13)	ns	-	-

**Table 5:** NGS mutations as assessed by IonTorrent technology in 7 patients with low miR125a-5p expression (in patients 4 and 6, 2 different clones, a and b, were observed)

pat	Gene	Chr	Position	Location	Ref sequence	Var sequence	Mutation Type	Freq. %
1	DDX3X	Chr	41.204.747	exonic	GAAAGTAGTTT GGGTGGAAGA	GAAAGTAGTT----TGGAAGA	Frame shift deletion	39,1
2	SF3B1	ch2	198.267.491	exonic	C	G	Non synonymous SNV	52,69
3	MYD88	chr3	38.182.641	exonic	T	C	stoploss SNV	10,83
4a	TP53	chr17	7.577.575	exonic	A	C	Non synonymous SNV	6,43
4b	TP53	chr17	7.577.580	exonic	T	C	Non synonymous SNV	18,89
5	XPO1	chr2	61.719.472	exonic	C	T	Non synonymous SNV	26,33
6a	ATM	chr11	108.138.003	exonic	T	C	Non synonymous SNV	64,06
6b	FBXW7	chr4	153.249.384	exonic	C	T	Non synonymous SNV	32,94
7	TP53	chr17	7.578.536	exonic	T	C	Non synonymous SNV	28,59

In a cohort of untreated low-risk CLL patients as defined by CD38 negativity (CD38+ cells < 7%) and low-risk FISH findings (normal or 13q deletion as single aberration), we therefore studied the biological and clinical significance of the presence of genetic heterogeneity in the minor CD38+ leukemic population.

Our data showed that a significant proportion of CD38- CLL patients with low risk FISH findings presented genetic aberrations within CD38+ cells. Most of these abnormalities were high risk lesions (11q deletion in 9 cases, 17p deletion in 8 cases) and in most of the cases these lesions were found in different cells indicating that multiple cytogenetically unrelated minor clones may be present in the CD38+ cell fraction. The small size of the abnormal C38+ clones precluded the detection of these aberrations when analyzing by FISH the entire neoplastic population consisting of a majority of CD38-cells. Interestingly, the presence of these additional FISH lesions in the small CD38+ cell fraction was associated with shorter TTFT.

Genetic complexity and heterogeneity in the architecture of the leukemic clone was previously documented in CLL and was associated with disease progression and shorter TTFT [18]. Moreover, Grubor et al [11] in 3 out of 4 CLL patients found genetic imbalances between CD38+ cells and CD38- cells, some of which involved loci of clinical relevance including ATM and TP53. However, it is noteworthy that our findings apply to a the subset of CLL patients with “favorable” prognostic features in terms of CD38 expression and cytogenetic data on the entire cell population.

To identify biomarkers associated with this phenomenon, we performed miRNA expression analysis because deregulation of miRNA was previously shown to be associated with activation markers [14]. By comparing C1 patients with and without small abnormal clones in the CD38+ fraction, we were thus able to show a deregulated miRNA expression profile in CLL cases with additional FISH lesions in CD38+ cells. In particular, miR-125a-5p was found to be down-regulated both in CD38+ and CD38- cells in patients with FISH abnormal clones as compared to patients without FISH abnormal clones. miR-125a-5p was therefore chosen as a marker associated with genetic complexity and possibly with a more aggressive clinical behavior as suggested by the shorter TTFT that we observed in these patients.

The relevance of miR-125a-5p as a biomarker of inferior outcome and genetic complexity was then validated in a prospective cohort of 71 consecutive untreated CD38- CLL patients with normal FISH or 13q deletion as single abnormality. In this validation cohort we were able to confirm the predictive role of miR-125a-5p down-regulation in terms of shorter TTFT. To our knowledge, this is the first observation linking a deregulated miRNA expression to inferior outcome in a subset of low risk CLL patients [19]. This finding is valuable because the majority of CLL patients present with low risk features at diagnosis and disease progression is difficult to predict in such cases.

In addition, in this validation cohort we found, through the use of NGS technology, that CLL patients with lower levels of miR-125a-5p displayed an increased rate of mutations in CLL-related genes. Several recent reports have correlated the presence of specific mutations, detected by NGS, to prognosis [9, 20]. Interestingly most of the mutations found in our patients, including TP53, SF3B1 and ATM, have been associated with a worse clinical outcome and prognosis. This observation further strengthens the association between genetic complexity, miR-125-5p down-regulation and worse outcome.

Alterations in miRNA expression are involved in the initiation, progression, and metastasis of several human cancers, by acting both as tumor suppressors and oncogenes in cancer development [21]. In particular, miR-125a-5p was previously found to act as a non-organ specific tumor suppressor gene that, when down-regulated, is associated, in several solid cancers, with a more aggressive course of the disease and a worse prognosis [22-26]. A germline mutation in mature miR-125a-5p has also been closely associated with breast cancer tumorigenesis [27]. This miRNA primarily achieves its antiproliferative effect through down-regulation of proliferation related genes, involved in the phosphoinositide-3 kinase (PI3K)-AKT and RAS/RAF/mitogen-activated protein kinase signaling [23]. Noteworthy, it was recently shown that in SMZL, an indolent B cell lymphoproliferative disorder like CLL, there is a characteristic deregulation of miRNA expression including down-regulation of miR-125a-5p with a possible implication in its molecular tumorigenesis [28]. By contrast, miR-125a was also found commonly gained and/or overexpressed in DLBCL [29] and in this perspective it would be interesting to look at miR-125a-5p levels in CLL patients that has transformed into Richter's syndrome.

In conclusion, (i) by FISH analysis we disclosed genetic lesions in the minor of CD38+ cell fraction in CD38- CLL with low-risk FISH findings, (ii) we provided evidence supporting an association between cryptic genetic lesions in CD38+ cells and disease progression, (iii) we found that genomic complexity and worse outcome were associated with miR-125a-5p down-regulation and, iv) we validated this finding in an independent cohort of untreated CD38- CLL patients with low risk FISH findings.

This set of data show that genetic lesions may appear in CD38+ cells in low-risk CLL and that they may be associated with miR-125a-5p down-regulation, allowing for a more accurate prognostication in low-risk CLL.

## METHODS

### Patients

In this study 2 cohorts of patients have been considered. Cohort one (C1) included 28 untreated CLL patients seen between 2005 and 2006. Cohort two (C2) consisted of 71 consecutive untreated CLL patients diagnosed between 2007 and 2011. Inclusion criteria were the following: diagnosis of CLL according to NCI criteria [30], CD38 negativity (CD38 percentage < 7%), good prognosis genetic lesions as defined as isolated 13q deletion or absence of genetic lesions by FISH, here referred to as FISH negativity, using a standard 4-probe FISH panel [31] (vide infra).

Indications for treatment included: increased WBC count with <6 month lymphocyte doubling time, anemia or thrombocytopenia due to bone marrow infiltration or autoimmune phenomena not responding to steroids, disease progression in the Binet staging system. Fludarabine-containing regimens were used as first-line treatment; chlorambucil was used in some elderly and unfit patients.

This study was approved by the local ethics committee and informed consent was obtained from the patients.

### Immunophenotypic analysis

At diagnosis, immunophenotypic analysis was performed according to NCI criteria [30] as previously described [32]. The Matutes immunophenotypic score [33] was calculated giving 1 point each to CD5 positivity, CD23 positivity, CD22 weak positivity, sIg weak positivity and FMC7 negativity. Only patients with a score ≥3 (i.e. typical CLL) were included. The expression of CD38 and ZAP-70 were tested, as described [34], on fresh peripheral blood (PB) cells with a 7% and 30% cut-off for positivity, respectively.

### Cell isolation by immunomagnetic sorting

In the 28 patients in C1, CD38+ and CD38- CLL cells were isolated by immunomagnetic sorting as previously described [35]. Briefly, PB mononuclear cells (PBMCs) were isolated by density gradient separation (Lympholyte-H Cedarlane, Cellbio. Milan Italy). In order to eliminate monocytes, T and NK cells and to obtain a cell fraction enriched in B-cells we first performed a negative selection with Dynabeads PanMouse IgG (Dynal A.S., Oslo, Norway) coated with anti-CD14, anti-CD3, and anti-CD16 monoclonal antibodies (clones UCHM1, UCHT1 and B-E16, respectively, all provided by Denamed, Ferrara, Italy). CD3, CD14 and CD16 negative cells (i.e. B cells) were subsequently subjected to a positive selection with Dynabeads coated with anti-CD38 antibody (clone T16, Denamed Ferrara, Italy). At the end of this procedure we obtained CD38+ and CD38- B cells. The purity of sorted CD38- and CD38+ B lymphocytes was > 98% as determined by flow cytometric analysis.

### FISH analysis

For CLL risk assessment, interphase FISH was performed in all patients on PB samples obtained at diagnosis using probes for the following regions: 13q14, 12q13, 11q22/ATM, 17p13/TP53 (Vysis/Abbott Co, Downers Grove, IL, USA) as described [32]. Cut-off points for positivity were previously reported [32].

In C1 patients, FISH analysis was performed on both CD38+ and CD38- immunomagnetic sorted cells, as previously described [35], and the following regions were investigated: 13q14, 12q13, 11q22/ATM, 17p13/TP53 and 14q32 (LSI IGH) (Vysis/Abbott Co, Downers Grove, IL, USA). The following probes were used as controls: CEP10, LSI PDGFRB, LSI RUNX1/RUNX1T1, LSI BCR/ABL (Vysis/Abbott Co, Downers Grove, IL, USA). Co-hybridization experiments were performed in order to evaluate the coexistence on the same cells of more genetic lesions. The sensitivity limits for FISH analysis on sorted cells were calculated on 5 normal healthy controls as median values + 3 standard deviations and were set at 10% for translocations and trisomies, and at 14% for deletions.

### *IGHV* and *TP53*

*IGHV* genes were amplified from genomic DNA and sequenced according to standard methods and the cut-off of 98% homology to the germline sequence was chosen to discriminate between mutated (<98% homology) and unmutated (≥98% homology) cases, as reported previously [8]. TP53 mutational analysis was performed as described elsewhere [8].

### MicroRNA microarray

MiRNA expression was investigated using the Agilent Human miRNA microarray v.2 (#G4470B, Agilent Technologies). as previously described [36]. Microarray results were analyzed by using the GeneSpring GX v.12 software (Agilent Technologies). Data transformation was applied to set all the negative raw values at 1.0, followed by a Quantile normalization and a log2 transformation. Filters on gene expression were used to keep only the miRNAs expressed (Detected) in at least one sample. Then, samples were grouped according to the presence or not of genetic lesions by FISH analysis in the CD38 positive cells. Differentially expressed miRNAs were identified by using a 2 fold-change filter followed by a moderated t-test, with p<0.05 using a moderated t-test with Benjamini-Hochberg correction. Microarray experiments have been submitted to ArrayExpress database (accession number to be received).

### Quantitative Real time RT-PCR (qRT-PCR)

Mature miRNAs expression was evaluated by Taqman MicroRNA assays (Applied Biosystem, Life Technologies, Foster City, CA, USA) specific for miR-125a-5p and normalized on 18S ribosomal RNA. Briefly, 5 ng of total RNA was reverse transcribed using the specific looped primer and quantitative real-time reverse transcription PCR (qRT-PCR) was conducted using the standard TaqMan MicroRNA assay protocol on a Bio-Rad-CFX thermal cycler (Bio-Rad Laboratories, Hercules, CA, USA). Each sample was analyzed in triplicate. qRT-PCR for 18S rRNA was performed using 500 ng of total RNA for each sample according to the instructions of the manufacturer (M-MLV Reverse Transcriptase; Promega, Madison, WI, USA) and the real-time reaction using EvaGreen® Dye (Biotium, Hayward, CA, USA) on the Bio-Rad-CFX instrument. Each sample was analyzed in triplicate. The level of miRNA and mRNA was measured by the use of the quantitation cycle (Cq). The amount of target, normalized on 18S rRNA amount, was calculated using 2-ΔCq (comparative Cq) method as implemented by Biorad CFX Manager Software (Bio-Rad Laboratories, Hercules, CA, USA). Significance in qRT-PCR results was determined by t-test.

### IonTorrent Personal Genome Machine (PGM) analysis

Agilent HaloPlex Target Enrichment kit (Agilent Technologies, Santa Clara, CA, USA) was used to construct libraries of spot exonic regions of 20 genes (ATM, BIRC3, BRAF, CDKN2A, CTNNB1, DDX3X, FBXW7, KIT, KLHL6, KRAS, MAPK1, MYD88, NOTCH1, NRAS, PIK3CA, POT1, SF3B1, TP53, XPO1, ZMYM3) starting from genomic DNA from peripheral blood samples, according to HaloPlex Target Enrichment System (Agilent Technologies, Santa Clara, CA, USA). Diluted libraries were linked to Ion Sphere Particles, clonally amplified in an emulsion PCR and enriched using Ion OneTouch emulsion PCR System (Life technologies, Foster City, CA, USA). Exon-enriched DNA was precipitated with magnetic beads coated with sptrptavidin. Enriched, template-positive Ion Sphere Particles were loaded in one Ion chip and sequenced using Ion Torrent PGM (Life technologies, Foster City, CA, USA). Sequencing data were aligned to the human reference genome (GRCh37). Data analysis and variants identification were performed using Torrent Suite 3.4 and Variant Caller plugin 3.4.4 (Life technologies, Foster City, CA, USA).

### Statistical analysis

Quantitative variables were reported as mean values with standard deviations (SDs) and were compared using the Mann-Whitney test. The Fisher exact test was used for categorical variables. All tests were 2-sided. The time to first treatment (TTFT) was calculated as the interval between diagnosis and the start of first-line treatment. Survival curves were compared by using the log-rank test. A P value <.05 was used as a criterion for statistical significance. Proportional hazards regression analysis was used to identify the most significant independent prognostic variables on TTFT. Statistical analyses were performed using Stata software release 8.0 (Stata Corporation, College Station, TX, USA) and Prism 4.00 for Windows, GraphPad Software (San Diego California USA).

### Authorship contributions

G.M.R., M.N. and A.C. created and designed the study; G.M.R., F. Cibien, F. Cavazzini, M.C., G.D, and O.S. provided study materials and patients; L.R. and M.C. performed the FISH analysis; E.S., L.R., L.L., C.B., B.Z. and M.F. performed the molecular studies; G.M.R. S.M., L.F., M.C, and E.L. collected and assembled data; all authors assisted in the analysis and/or interpretation of the data; all authors critically revised the manuscript; and all authors gave final approval of the manuscript.

## Supplementary Figures and Tables




