# Supplementary material for: Genetic subclonal complexity and miR125a-5p down-regulatio identify a subset of patients with inferior outcome in low-ris CLL patients
Source: Oncotarget. 2013 Oct 30;5(1):140–9. doi: 10.18632/oncotarget.1382 (PMC3960196; doi:10.18632/oncotarget.1382)
Supplement: Supplementary file 1 [file oncotarget-05-0140-s001.pdf]

Genetic subclonal complexity and miR125a-5p down-regulation identify a subset of patients with inferior outcome in low-risk CLL patients - Rigolin et al

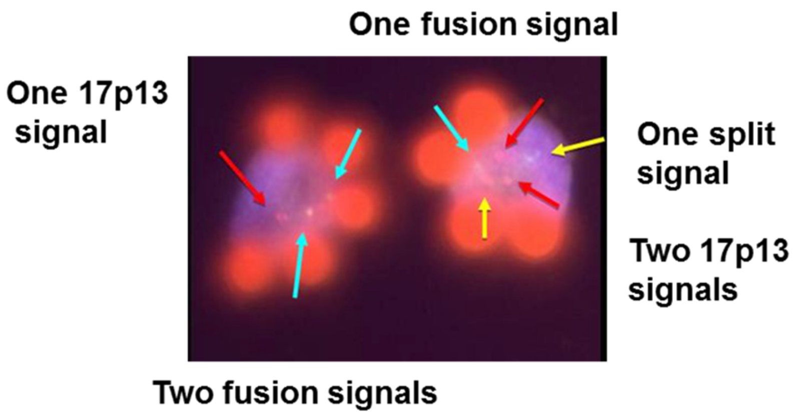

Supplemental Figure 1: Co-hybridization FISH analysis on CD38+ immunomagnetic sorted cells in case No. 61 using a 14q32 probe and a 17p13 probe showing that 17p deletion and 14q32 rearrangements are on different cells. The normal hybridization pattern for 17p13 probe is represented by 2 red signals (two red arrows), and for 14q32/IgH probe by 2 fusion signals (two blue arrows). On the left a CD38+ cell is shown with two fusion signals (two blu arrows) documenting the absence of 14q32 translocation, and one red signal (one red arrow) documenting the presence of 17p deletion. On the right it is shown a cell with 2 red signals (absence of 17p deletion, two red arrows) and one split signal (two yellow arrows) indicating the presence of 14q32 translocation. Note that several Dynabeads are attached to the cells.

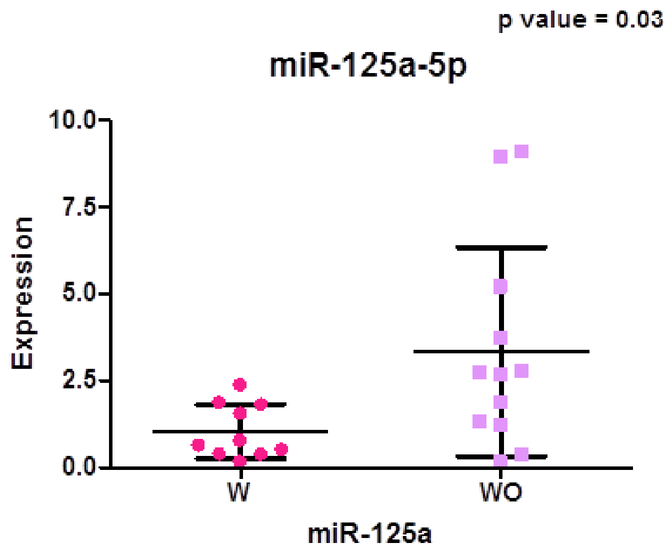

Supplemental Figure 2: RT-qPCR validation of miR125a-5p expression according to the presence (W: mean value 1.043 +/- 0.246, n=10) or not (WO; mean value 3.338 +/- 0.868, n=12: p=0.03) of FISH lesions in CD38+ cells.
